# Probe-based multiplex qPCR identifies blood-meal hosts in *Anopheles* mosquitoes from Papua New Guinea

**DOI:** 10.1186/s13071-020-3986-6

**Published:** 2020-02-28

**Authors:** John B. Keven, Georgia Artzberger, Mary L. Gillies, Rex B. Mbewe, Edward D. Walker

**Affiliations:** 1 Department of Microbiology and Molecular Genetics, Michigan State University, East Lansing, MI USA; 2 Vector Borne Diseases Unit, Papua New Guinea Institute of Medical Research, Madang, Papua New Guinea; 3 Department of Biomedical Laboratory Diagnostics, Michigan State University, East Lansing, MI USA; 4 Department of Entomology, Michigan State University, East Lansing, MI USA; 5 Department of Physics and Biochemical Sciences, University of Malawi, The Polytechnic, Blantyre, Malawi

**Keywords:** *Anopheles*, Blood meal, Host, Mosquitoes, Multiplex

## Abstract

**Background:**

Determination of blood-meal hosts in blood-fed female *Anopheles* mosquitoes is important for evaluating vectorial capacity of vector populations and assessing effectiveness of vector control measures. Sensitive molecular methods are needed to detect traces of host blood in mosquito samples, to differentiate hosts, and to detect mixed host blood meals. This paper describes a molecular probe-based quantitative PCR for identifying blood-meal hosts in *Anopheles* malaria vectors from Papua New Guinea.

**Methods:**

TaqMan oligonucleotide probes targeting specific regions of mitochondrial or nuclear DNA of the three primary *Anopheles* blood-meal hosts, humans, pigs and dogs, were incorporated into a multiplex, quantitative PCR which was optimized for sensitivity and specificity.

**Results:**

Amplification of serially diluted DNA showed that the quantitative PCR detected as low as 10^−5^ ng/μl of host DNA. Application to field-collected, blood-fed *Anopheles* showed that the quantitative PCR identified the vertebrate hosts for 89% (335/375) of mosquitoes whereas only 55% (104/188) of blood-meal samples tested in a conventional PCR were identified. Of the 104 blood-fed *Anopheles* that were positive in both PCR methods, 16 (15.4%) were identified as mixed blood meals by the quantitative PCR whereas only 3 (2.9%) were mixed blood meals by the conventional PCR.

**Conclusions:**

The multiplex quantitative PCR described here is sensitive at detecting low DNA concentration and mixed host DNA in samples and useful for blood-meal analysis of field mosquitoes, in particular mixed-host blood meals.

## Background

Identification of blood-meal hosts in blood-sucking arthropod vectors of human diseases forms an integral component of analysis of both vectorial capacity and effectiveness of vector control measures. For human malaria, analysis of female *Anopheles* mosquito blood meals is important for assessing the degree of feeding on humans (anthropophagy), usually expressed in terms of human blood index or HBI, i.e. the proportion of blood-engorged mosquitoes that fed on humans relative to non-human hosts. This index is used to evaluate epidemiological outcomes of vector-targeted malaria control programmes because vectorial capacity, which is a measure of disease transmission potential of a vector population, increases with the HBI [1, 2]. Blood-meal analysis is also important for assessing host selection, which is the propensity of a mosquito population to feed more or less on a host species relative to other host species available to the mosquito [3]. Assessment of host selection is important when considering vector control methods such as insecticide-treated bednets and zooprophylaxis which may divert mosquito feeding to non-human hosts.

Immunological methods such as precipitin test, latex agglutination and enzyme-linked immunosorbent assay (ELISA) were the earliest methods used in mosquito blood-meal analysis [4–7]. These methods have produced countless valuable data [3, 8–13] but have been superseded in recent years by nucleic acid-based methods. In cases where mosquito populations are known to feed on a wide range of vertebrate hosts or when the host range has not been characterized, sequencing of amplicons amplified with generic primers followed by BLAST (Basic Local Alignment Search Tool) search against publicly available vertebrate host sequences has been used [14–18]. However, this approach is expensive and sequencing facilities are not available in many laboratories. In cases where mosquito populations are known to feed on a limited range of host species, less expensive methods such as restriction-fragment length polymorphisms [19], DNA-DNA hybridization [20], conventional multiplex PCR with species-specific primers [21] or quantitative PCR (qPCR) with species-specific probes [22, 23] are employed.

In Papua New Guinea (PNG), the blood-meal host range for the *Anopheles* vectors of malaria is limited to humans, pigs and dogs [9, 10, 16, 24]. Thus, PCR methods with host-specific primers or probes are appropriate in this setting. Conventional multiplex PCR with primers specific to these three hosts is currently applied to mosquitoes originating from PNG [24]. However, with a large sample, the gel-electrophoresis step can be labor-intensive and time-consuming, resulting in accumulation of unanalyzed samples and delays in data availability. Furthermore, as shown in this present study, the conventional multiplex PCR has a high rate of false-negative blood meals and is less sensitive at detecting mixed blood meals. The objective of this study was to develop a probe-based multiplex qPCR for identifying the common mammalian blood-meal hosts in female *Anopheles* mosquitoes originating from PNG.

## Methods

### Samples

Genomic DNA isolated directly from human and animal tissues was used in the optimization of the qPCR. Pig DNA was isolated from a piglet’s tail (25 mg), removed as part of standard tail-docking husbandry routine at the Michigan State University Swine Farm. Dog DNA was isolated from a blood sample (50 μl) by a laboratory that studies genetic diseases of dogs. Human DNA was isolated from a blood sample (50 μl) of a study participant in PNG during a cross-sectional malaria epidemiology survey by PNG Institute of Medical Research. Genomic DNA isolated from abdomens of blood-fed *Anopheles* mosquitoes collected in a malaria-endemic village of PNG was used to test the new qPCR method. DNA extractions were performed using DNeasy Blood & Tissue Kit (Catalog number: 69506; Qiagen, Valencia, CA, USA).

### Probe design

Nucleotide sequences for the primers and probes described in this study are shown in Table 1. The probes were designed within the mitochondrial cytochrome *c* oxidase subunit 1 gene (*cox*1) for pig, mitochondrial cytochrome *b* gene (*cytb*) for dog and intron 1 of tyrosine hydroxylase gene (*th*) for human using the online program PrimerQuest accessible at the Integrated DNA Technologies website. Each probe was labelled at the 5′ end with reporter dye FAM, VIC or ABY and at the 3′ end with TaqMan quencher dye QSY (see Table 1). The primers and probes were obtained from Invitrogen and Applied Biosystems, respectively through Thermo Fisher Scientific (Waltham, MA, USA).

**Table 1:** Primers and probes for the qPCR method for three vertebrate host species

Organism	Primer/probe name	Nucleotide sequence (5′-3′)	Amplicon size (bp)
Human	humanTH1_F	GGCCTGTTCCTCCCTTATTT	101^a^
	humanTH1_R	TACACAGGGCTTCCGAGT	
	humanTH1_P	FAM-ATGGAGTCTGTGTTCCCTGTGACC-QSY	
Pig	pigCOX1_F	CTGACTACTTCCACCATCCTTC	108
	pigCOX1_R	TGGGCTAAGTTTCCAGCTAAA	
	pigCOX1_P	VIC-ATAGTAGAAGCCGGAGCGGGTACT-QSY	
Dog	dogCYTB_F	TGGACAAAGCAACCCTAACA	103
	dogCYTB_R	CCGGTTTCGTGTAGAAATAGGA	
	dogCYTB_P	ABY-TCATCCTCCCTTTCATCATCGCAGC-QSY	

^a^Due to presence of a microsatellite repeat sequence (5′-AATG-3′) within the human amplicon, the PCR product size varies between different human individuals. Presence of the repeat sequence does not affect PCR amplification as the primers and probe sequence do not extend into the repeat region

### Development of multiplex qPCR

For each target organism, the optimum qPCR reaction mixture was determined by testing different concentrations of the specific probe and primers in uniplex reactions (10 μl volume) containing 1× TaqMan Multiplex Master Mix (Catalog Number: 4461882; Thermo Fisher Scientific, Waltham, MA, USA) and 2 μl of serially diluted (10-fold dilutions) template DNA samples (10 ng/μl starting concentration). PCR reactions were performed on QuantStudio 7 Flex instrument (Applied Biosystems, Foster City, CA, USA) with Fast Cycling condition consisting of one cycle of 95 °C for 20 s followed by 40 cycles of 95 °C for 1 s and 60 °C for 20 s. Results were evaluated in software QuantStudio (version 1.3). Standard curves were evaluated and the reagent concentration that gave the highest percent amplification efficiency (measured as Efficiency = (10 ^(−1/slope)^ − 1) × 100) and lowest threshold cycle or C_q_ (which corresponds to highest sensitivity) was considered optimum and was used as the starting concentration for optimization of multiplex qPCR. For each of the three assays, multiplex qPCR mixture that gave the highest sensitivity and percent efficiency was considered optimum. The standard curve for each assay was derived from amplification results of triplicate dilution series (10-fold) of the target DNA.

### Assay validation

Specificity of the probes and primers to their target DNA region was confirmed *in silico* by performing BLAST and primer-BLAST [25] searches for potential matches to untargeted sequences in the National Center for Biotechnology Information database. Cross-reactivity of each probe to non-target organisms was also tested *in vitro* by subjecting DNA of the non-target organisms to uniplex qPCR containing the probe. For example, the human probe was tested for cross-reactivity to eight vertebrate species, namely pig, dog, cow, goat, horse, cat, rat and chicken; three *Plasmodium* species likely to be present in the blood meals, namely *P. falciparum*, *P. vivax* and *P. malariae*; and five species of *Anopheles* mosquitoes commonly found in PNG, namely *An. farauti* (*sensu stricto*), *An. koliensis*, *An. punctulatus* (*s.s.*), *An. longirostris* and *An. bancroftii*.

Ten-fold serially diluted DNA samples of humans, pigs and dogs were amplified in the multiplex qPCR and the popular conventional, multiplex PCR [21] and their results were compared for sensitivity. Primers for the single gene target of the mitochondrial *cytb* gene, reaction mixture and cycling condition for the conventional PCR used in this study was as previously described [24]. The *Anopheles* blood-meal DNA samples (*n* = 375) were analyzed in the qPCR to validate the applicability of the method to field mosquitoes. A subset of these qPCR-tested blood-meal samples (*n* = 188) was also analyzed in conventional PCR and its amplification success rate was compared with that of the qPCR. Amplification results with C_q_ value ≥ 38 were inconclusive and therefore considered negative.

For mosquitoes whose blood-meal hosts were successfully identified by the qPCR, the host DNA concentration were estimated using the general formula

$$log_{10} \left( {Conc} \right) = \left( {C_{q} - Intercept} \right)/Slope$$

where *Conc* is the host DNA concentration in ng/μl. The slope and intercept are specific to each host species (see Table 2). For the single blood-meal mosquitoes, the mean host DNA concentration for those that were detected in the conventional PCR was statistically compared (Studentʼs two-sample t-test) with those that were undetected. Similarly, for the mixed blood meals, the mean DNA concentration for the hosts that was detected in the conventional PCR was compared with those that were undetected.

**Table 2:** Standard curve and reaction efficiency of the three assays

Organism	Slope	% Efficiency	Y-intercept	*R* squared	SE
Human	− 3.36	98.4	25.6	0.997	0.21
Pig	− 3.50	93.1	22.3	0.998	0.19
Dog	− 3.51	92.7	23.7	0.996	0.22

*Abbreviation*: SE, standard error

## Results

**Fig. 1:**
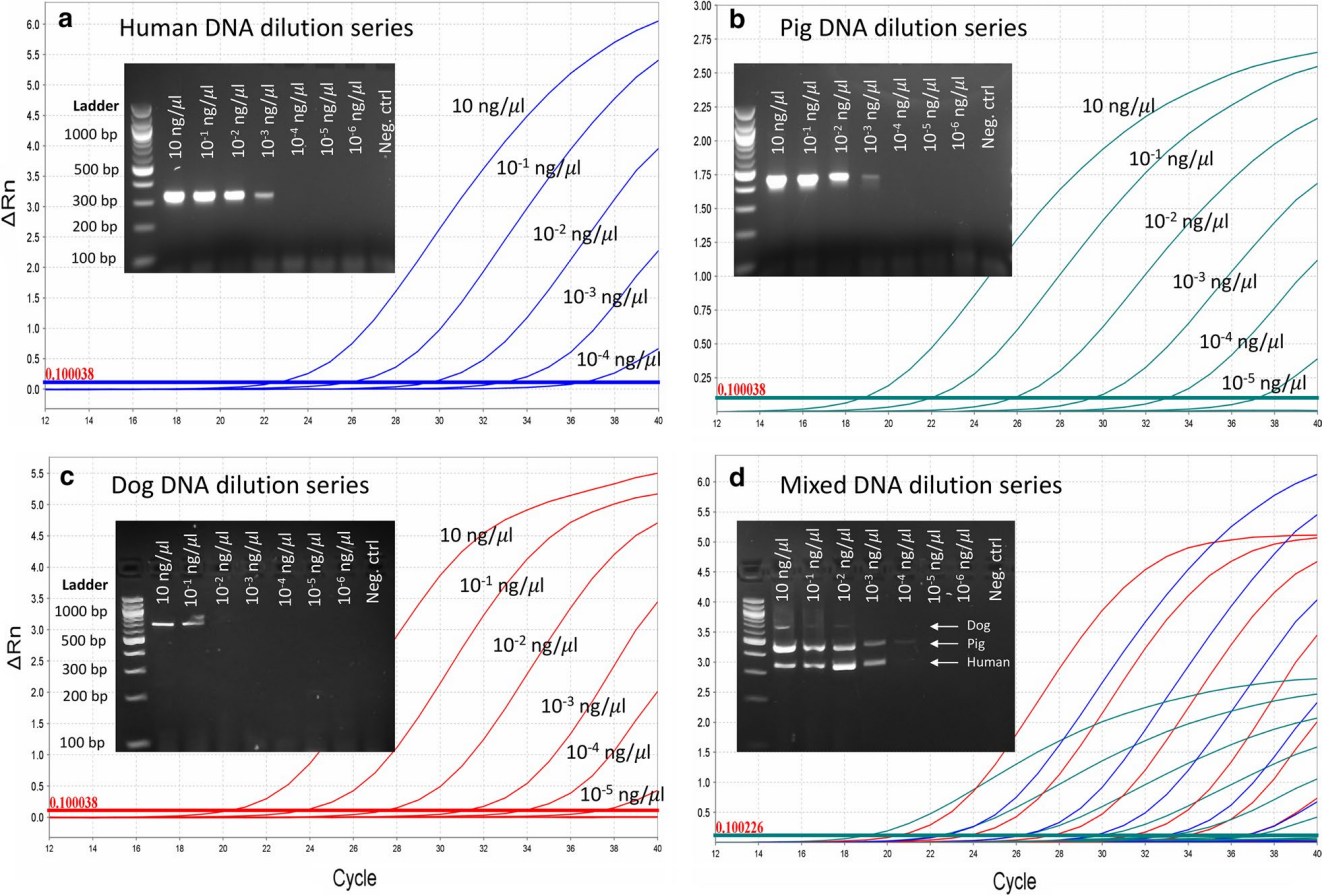
Results of conventional (i.e. the agarose gel images) and quantitative (i.e. the linear amplification plots) multiplex PCR both applied to the same 10-fold DNA dilution series for human (**a**), pig (**b**), dog (**c**) and mixed sample (**d**). For the qPCR curves in (**d**), color blue indicates human, green indicates pig and red indicates dog as in (**a-c**). The mixed samples (**d**) contain equal DNA concentration (in ng/μl) of each host

The three blood-meal probes performed well together in multiplex reactions and were specific to their target organism. Optimum reaction mixture for the multiplex qPCR (10 μl volume) consisted of 1× TaqMan Multiplex Master Mix, 0.6 μM of each human primer, 0.5 μM of each dog and pig primer, 0.3 μM of human probe and 0.25 μM of pig and dog probes and DNA template (concentration vary by samples). Standard curves and reaction efficiencies for the three assays are shown in Table 2. The assays were sensitive at detecting low DNA concentrations; the lowest detectable DNA concentration in the dilution series was 10^−4^ ng/μl for human and 10^−5^ ng/μl for pig and dog (Fig. 1a–d). In contrast to the qPCR, the conventional one did not detect DNA concentrations below 10^−3^ ng/μl for human, 10^−4^ ng/μl for pig and 10^−2^ ng/μl for dog (Fig. 1a–d). In addition to the insensitivity of the dog primers to low DNA concentration, amplification of the higher DNA concentration resulted in weak PCR bands compared to that of human and pig (Fig. 1c, d). Also, for all three hosts, the PCR band intensity in mixed DNA samples (Fig. 1d) was weaker than single host DNA samples (Fig. 1a–c).

Of the 375 *Anopheles* blood meals analyzed in the qPCR, 335 (89.3%) were positive for one or more of the three vertebrate hosts whereas only 40 (10.7%) were negative. Of the 335 positive samples, 225 (67.2%) were human blood meals, 18 (5.4%) were pigs, and 41 (12.2%) were dogs, with no other hosts detected in these single host results. The remaining 15.4% of positive samples were of mixed species, including 11 (3.3%) human + pig, 32 (9.55%) human + dog, four (1.2%) pig + dog, and four (1.2%) human + pig + dog. A random subset of these blood-meal samples (*n* = 188) was analyzed in the conventional, multiplex PCR and the results were compared with that of the qPCR by means of concordance-discordance frequency matrix (Table 3). Of 104 samples that were positive in both PCR methods, 16 (15.4%) were identified as mixed blood meals by the qPCR whereas only 3 (2.9%) were mixed blood meals by the conventional PCR. Twenty-four samples that were negative in the qPCR were also negative in the conventional PCR (i.e. 100% concordance). For those blood meals that were positive in the qPCR reactions (columns of Table 3), only 62.3% of human, 63.6% of pig, 50% of dog, 0% human + pig mix, 20% of human + dog mix and 0% of pig + dog mix were concordant with conventional PCR results. The majority of the discordant blood meals were mosquitoes that were positive by the qPCR but were negative in the conventional PCR (*n* = 60); only a few (*n* = 16) were actually incongruent in the blood-meal types determined by the two methods. Of these, 13 were mosquitoes with mixed blood meals by qPCR but appeared as single blood-meal types in the conventional PCR (Table 3).

**Table 3:** Frequency of concordant and discordant *Anopheles* blood-meal results analyzed by quantitative *versus* conventional PCR

Conventional PCR	Quantitative PCR						
	Human	Pig	Dog	Human/pig	Human/dog	Pig/dog	Negative
Human	66 (62)	0 (0)	2 (8)	2 (33)	9 (60)	0 (0)	0 (0)
Pig	1 (1)	7 (64)	0 (0)	1 (17)	0 (0)	0 (0)	0 (0)
Dog	0 (0)	0 (0)	12 (50)	0 (0)	1 (7)	0 (0)	0 (0)
Human/pig	0 (0)	0 (0)	0 (0)	0 (0)	0 (0)	0 (0)	0 (0)
Human/dog	0 (0)	0 (0)	0 (0)	0 (0)	3 (20)	0 (0)	0 (0)
Pig/dog	0 (0)	0 (0)	0 (0)	0 (0)	0 (0)	0 (0)	0 (0)
Negative	39 (37)	4 (36)	10 (42)	3 (50)	2 (13)	2 (100)	24 (100)

*Notes*: Numbers outside of parenthesis are frequency of concordant (along diagonal) or discordant (off-diagonals) blood-meal types and inside parenthesis are percentage of column-wise total

**Fig. 2:**
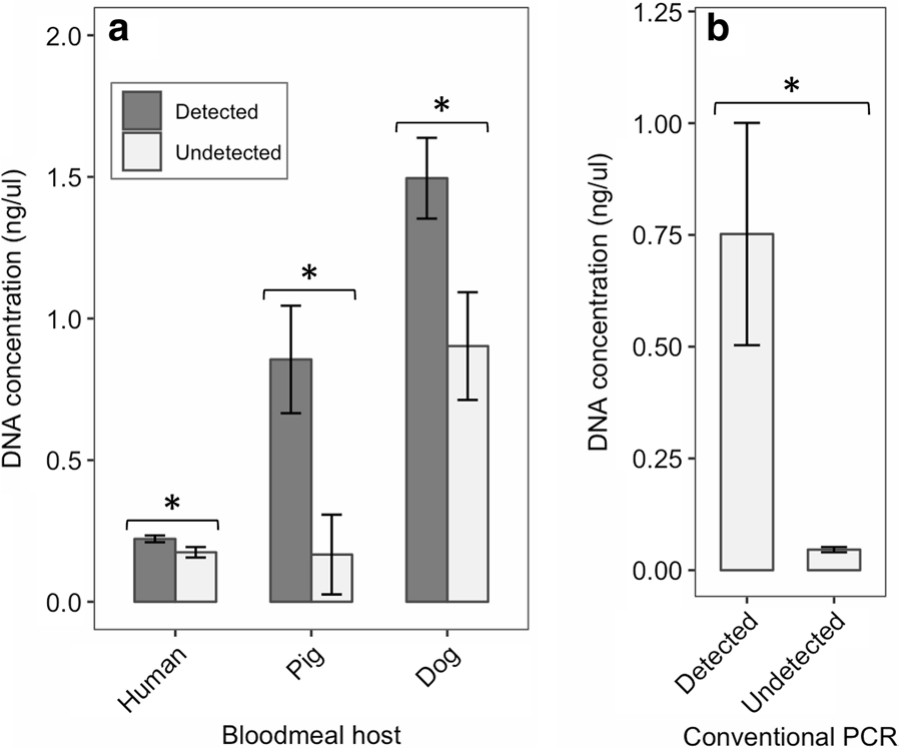
Bar plot showing mean DNA concentration (± standard error bar) for mosquito blood meals that were detected *versus* undetected by conventional PCR. **a** Comparison of mean DNA concentration for single blood meals grouped according to human (detected, *n* = 6; undetected, *n* = 38), pig (detected, *n* = 7; undetected, *n* = 3) and dog (detected, *n* = 12; undetected, *n* = 10) hosts. **b** Comparison for mixed blood meals (detected, *n* = 19; undetected, *n* = 22) not grouped according to host species. Asterisk (*) indicates significant difference (*P* < 0.05) between groups (Studentʼs t-test)

The results of the statistical comparison of the mean host DNA concentration of mosquito blood meals that were detected *versus* undetected in the conventional PCR are shown in Fig. 2 (see Additional file 1: Table S1 and Additional file 2: Table S2 for the data used in the statistical analyses). As predicted, for mosquitoes with a single blood-meal host (grouped according to host species), the mean DNA concentration of those that were detected was significantly higher than the undetected ones for human (t-test: *t*_(68)_ = 2.15, *P* = 0.04), pig (t-test: *t*_(8)_ = 2.9, *P* = 0.02) and dog (t-test: *t*_(17)_ = 2.49, *P* = 0.02) (Fig. 2a). Similarly, for mosquitoes with a mixed blood-meal (not grouped according to host species), the mean DNA concentration for those that were detected was significantly higher than the undetected ones (t-test: *t*_(18)_ = 2.84, *P* = 0.01) (Fig. 2b). The mean DNA concentration for detected single blood meals (Fig. 2a) was significantly higher for pig compared to human (Tukeyʼs HSD test: mean difference = 0.63, 95% family-wise confidence interval or CI: 0.40–0.86, adjusted *P* < 0.0001), higher for dog compared to human (mean difference = 1.27, 95% family-wise CI: 1.09–1.45, adjusted *P* < 0.0001) and higher for dog compared to pig (mean difference = 0.64, 95% family-wise CI: 0.36–0.9, adjusted *P* < 0.0001). For undetected single blood meals, the mean DNA concentration (Fig. 2a) were not significantly different between pig and human (mean difference = − 0.008, 95% family-wise CI: − 0.42–0.40, adjusted *P* = 0.999) but were significantly higher for dog compared to human (mean difference = 0.73, 95% family-wise CI: 0.48–0.97, adjusted *P* < 0.0001) and for dog compared to pig (mean difference = 0.74, 95% family-wise CI: 0.28–1.18, adjusted *P* = 0.0007).

## Discussion

The qPCR method offers several advantages over conventional PCR. First, when analyzing large number of samples with conventional PCR, the post-PCR steps such as gel-electrophoresis and manual scoring of results are labor-intensive, delay availability of data, generate large quantities of waste, and increase risk of exposure to ethidium bromide, a mutagen. These concerns are eliminated when qPCR is used. Secondly, it may be desirable to quantify DNA template or copy number of the target gene in samples, which qPCR can accomplish. Thirdly, while qPCR is not always more sensitive than conventional PCR [26], many studies including this current one have shown that the former method can detect much lower DNA template concentrations compared to the latter [27–30]. Thus, sensitivity of detection is better, as was shown here.

Identification of arthropod blood meals by qPCR has been applied to sand flies, biting midges, kissing bugs, fleas and mosquitoes [22, 23, 31–35]. Most of these were SYBR green-based systems; only three were probe-based. Of the three probe-based qPCR, one was for identifying Australian mammals in *Culex* mosquito blood meals and did not include humans, pigs or dogs [23], another was for identifying blood-meal hosts of biting midges and included humans and pigs but not dogs [33] and another was for identifying flea blood meals and included humans and dogs but not pigs [35]. Notably, the human probe in the latter flea blood-meal study was tested here and found to cross-react with dog DNA. Surprisingly, a thorough Google Scholar search did not find a paper describing probe-based qPCR designed specifically for identifying mammalian hosts of *Anopheles* blood meals. The qPCR assay described in the present study utilized the new non-fluorescent quencher dye QSY (Catalog number: 4482777; Applied Biosystems, Foster City, CA, USA) and a PCR solution optimized for probe-based multiplex qPCR (TaqMan Multiplex Master Mix, Catalog number: 4461882; Applied Biosystems) to detect the common blood-meal hosts of PNG mosquitoes.

When evaluating the sensitivity of the qPCR assay from amplifications of 10-fold dilution series of target DNA samples, the lowest detectable concentration for human DNA (10^−4^ ng/μl) was ten-fold greater than for pigs and dogs which was 10^−5^ ng/μl. This difference could be attributed to the copy numbers of the target DNA sequences; the pig and dog probes target mitochondrial genes which exist in multiple copies per cell, whereas the human probe targets a single-copy nuclear DNA sequence. Several human probes targeting various mitochondrial gene locus were designed and tested (see Additional file 3: Table S3). However, they all exhibited non-specific amplification of the two non-human hosts *in vitro* despite appearing to be target specific by *in silico* test. Nevertheless, the detectable limit of human DNA concentration with the current probe (10^−4^ ng/μl) is sufficiently low for detecting mosquito blood meals. The lower detectable limit of qPCR compared to the conventional one, particularly dog, indicates a difference in the sensitivity of the two methods.

The results show that the blood-meal qPCR was more sensitive at detecting host DNA in mosquitoes (detection success rate of 89%) compared to the more commonly used conventional, multiplex PCR (detection success rate of 55%). It is possible that the 11% of mosquitoes whose blood-meal hosts were not identified by the qPCR could have fed on other host sources (e.g. chickens, cats). However, when subjected to two conventional PCR utilizing generic mammalian and avian primers, none showed a positive result, which was consistent with findings from our previous study [24]. Thus, the likelihood that host breadth was greater than the three hosts we targeted with probes here is low. A common observation in all of these unamplified blood meals was that they all contained traces of blood in their abdomens (< 0.3 μl), based on light microscopy examination of the mosquito abdomens 4–8 hours after they were collected. Given the non-nucleated status of mammalian red blood cells and disproportionately low ratio of white to red blood cells, the small volumes of blood meal were likely insufficient to yield a detectable concentration of DNA.

The trace blood assertion was further supported by showing that the qPCR-quantified host DNA concentration of single blood meals was significantly higher in those samples that were detected in the conventional PCR than those that were undetected for all three hosts. The mean DNA concentration for the detected samples was expected to be statistically the same between the three hosts. However, the result showed otherwise; pig DNA concentration was significantly four-folds higher than humans and two-folds less than dog (Fig. 2a). This heterogeneity in host DNA quantity could be caused by factors such as variation among the hosts in the number of white blood cells per unit volume of blood. However, a more plausible explanation is variation in the average quantity of blood mosquitoes obtain from each host as a result of variation in host sensitivity to mosquito bites. That is, humans are more sensitive than pigs followed by dogs to mosquito bites, causing them to quickly interrupt blood-feeding mosquitoes before they had time to acquire the maximum amount of blood. Despite the variation between the three hosts in their blood-meal DNA quantity, the mean DNA concentration for mosquitoes that was undetected in the conventional PCR was expected to be the same. The expectation is based on the reasoning that below a certain DNA concentration threshold, the conventional PCR primers for all three hosts become insensitive to DNA and fail to amplify. However, the result showed that although the mean DNA concentration was statistically the same for humans and pigs as predicted, it was five-folds higher for dog (Fig. 2a). This indicates that the dog conventional primers are less efficient and have higher sensitivity threshold than the other two hosts, which is consistent with its behavior observed in the amplification of DNA dilution series.

The conventional PCR did not detect mixed blood meals sensitively; most of the samples identified as mixed blood meals by qPCR were identified as single blood meals by the conventional method (Table 3). The inaccurate detection of mixed blood meals was attributed to low DNA quantity of one or more of the hosts in a mixed blood-meal sample and was supported by the result which showed statistically lower mean DNA concentration for undetected compared to detected hosts (Fig. 2b). Thus, a primary outcome of this study is the sensitivity of the probe-based, qPCR method to detect blood from different mammal species in the same blood meal. This finding indicates that a significant proportion of unidentified blood-meal sources in studies that used the conventional, multiplex PCR [24, 36–41] may, among other factors, be due to the sensitivity of this method. Furthermore, the proportion of mixed blood meals may be underestimated and single blood meals overestimated in some published studies. At the very least, such findings indicate interrupted blood feeding, an important variable contributing to transmission [42].

## Conclusions

This paper describes the optimization of a probe-based multiplex qPCR for identifying the common blood-meal hosts of *Anopheles* mosquitoes from PNG. The qPCR assay was sensitive at detecting trace amounts of target DNA in samples compared to the standard PCR, which makes it appropriate for analysis of mosquito blood meals that often contain trace amounts of host blood. Its ability to detect mixed blood meals compared to the standard PCR, makes it appropriate for use in studies that investigate interrupted feeding habits of mosquitoes. Although the qPCR assay described here was intended for analysis of mosquito blood meals from PNG and other parts of South-West Pacific, addition of probes specific to vertebrate hosts of mosquitoes from other parts of the world (e.g. cows and goats in Africa) is possible and encouraged.

## Supplementary information

**Additional file 1: Table S1.** Data on conventional PCR host detection status, qPCR C_q_ value and host DNA concentration for mosquitoes with single bloodmeal.

**Additional file 2: Table S2.** Data on conventional PCR host detection status, qPCR C_q_ value and host DNA concentration for mosquitoes with mixed bloodmeals.

**Additional file 3: Table S3.** Human oligonucleotide probes targeting various mitochondrial gene locus that were specific to humans by *in silico* test but amplified pig or dog DNA by *in vitro* test.

## Data Availability

Data supporting the conclusions of this article are included within the article and its additional file.

## Abbreviations

BLAST: Basic Local Alignment Search Tool
DNA: deoxyribonucleic acid
ELISA: enzyme-linked immunosorbent assay
PCR: polymerase chain reaction
PNG: Papua New Guinea
qPCR: quantitative polymerase chain reaction
